# Mini-Mental State Examination not optimal for cognitive deficits in elderly heart failure patients

**DOI:** 10.1590/1806-9282.20242062

**Published:** 2025-06-02

**Authors:** Haibo Xu, Baogui Wang

**Affiliations:** 1 Shandong Women's University, School of Healthy Aging – Jinan, China.

Dear Editor,

We read a recent publication by Silva et al.^1^ that compared the Mini-Mental State Examination (MMSE) and Montreal Cognitive Assessment (MoCA) test for the identification of cognitive deficits (CDs) in elderly heart failure (HF) patients. The study employed a cross-sectional design in which the MMSE and MoCA CD test were administered to 43 elderly HF patients with a mean age of 67 years by using a clinical questionnaire on sociology. The data collected were analyzed using R Studio software, and Fisher's exact test was used to determine the differences between the MMSE and MoCA test, although the authors arrived at the conclusion that the MMSE performed better than the MoCA test in the identification of CDs in elderly patients with HF through their analysis. However, we believe that this study was not rigorous. Therefore, the authors must resolve the following issues to improve rigor.

First, there is the issue of the participants’ level of education. Although the authors clearly describe in their article that the test results were adjusted for the individual's level of education, such as limitations on scores for different years of education and the corresponding bonus points, this was fatal to the conclusion. The references used by the authors also mention that the MoCA test is not only inaccurate in detecting cognitive impairment no dementia (CIND) in people with low levels of education^2^ but also has a higher sensitivity to cognitive decline in patients with higher levels of education^3^. This suggests that the MoCA test can vary depending on the level of education. In contrast, the present study analyzed groups with different levels of education together, and although the results of the study showed a statistically significant difference between the MMSE and MoCA test (p=0.045), there was also a risk of false-positive results. Therefore, the authors need to differentiate the educational level of the participating population and conduct the study separately.

Second, the issue of reliability and internal consistency of MMSE and MoCA test. The authors mentioned in the "Methods" section that the MMSE has strong reliability and internal consistency in Brazil and did not retest the information of the data collected in the present exercise. However, this is inappropriate from the point of view of data analysis rigor; after all, when the authors conducted this study, the study population, the environment, and the time of the measurements, among other things, would have changed. In addition, the authors did not make any statement about the reliability and internal consistency of the MoCA test in the article. Therefore, we believe that to ensure the rigor of the scientific paper, the authors should consider testing the reliability and validity of the newly collected data in the article.

Finally, research design flaws. The article shows from the research objectives and conclusions that the authors chose to compare two scales, the MMSE and MoCA, by selecting the test that is more in line with the CDs of elderly HF patients. We believe that the article's use of a cross-sectional study design is not appropriate because a cross-sectional study is more focused on a certain time. At the level of the HF patient, the development of CDs can vary with the condition, rather than being fixed at a certain point in time. Therefore, the authors may consider conducting a longitudinal study or multiple cross-sectional studies at different time points to obtain results that are more applicable to HF patients at different stages of the disease.

Overall, although the authors demonstrate the conclusion that the MMSE is superior to the MoCA test in identifying CDs in elderly patients with HF, if the authors do not solve the issues raised in this paper well, we believe that the reliability and utility of the present conclusions are questionable. Therefore, the authors must address the issues raised in the above discussion to make the conclusions more rigorous.
